# An unusual case of obstructive uropathy

**DOI:** 10.1002/ccr3.4569

**Published:** 2021-08-16

**Authors:** Antigoni Xenou, Konstantinos A. Boulas, Maria Nathanailidou, Eytyxia Kyriakidou, Aikaterini Paraskeva, Alexandros Triantafyllidis, Konstantinos Chatzipourganis, Anestis Hatzigeorgiadis

**Affiliations:** ^1^ Department of General Surgery General Hospital of Drama Drama Greece

**Keywords:** hernioplasty, incarceration, inguinal hernia, obstructive uropathy, urinary bladder

## Abstract

Although inguinal bladder hernia associated with obstructive uropathy is an extremely rare entity, it should be suspected in elderly patients with bladder outlet obstruction presented with inguinal hernia and lower urinary tract symptoms.

## Graphical abstract

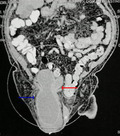

A 88‐year‐old male with history of severe benign prostatic hyperplasia with underactive bladder under permanent Foley catheter due to poor response to TURP presented with symptoms of catheter‐related urinary tract infection. Physical examination revealed right incarcerated inguinoscrotal hernia with the right testicle within the scrotum separable from the hernia and without findings of bowel obstruction. Kidney function tests suggested stage‐III acute kidney injury (serum creatinine 4.8 mg/dl with a 3.5 times increase from baseline). Renal ultrasonography revealed moderate bilateral hydronephroureterosis. CT performed to establish diagnosis (Figure 1).

**FIGURE 1:**
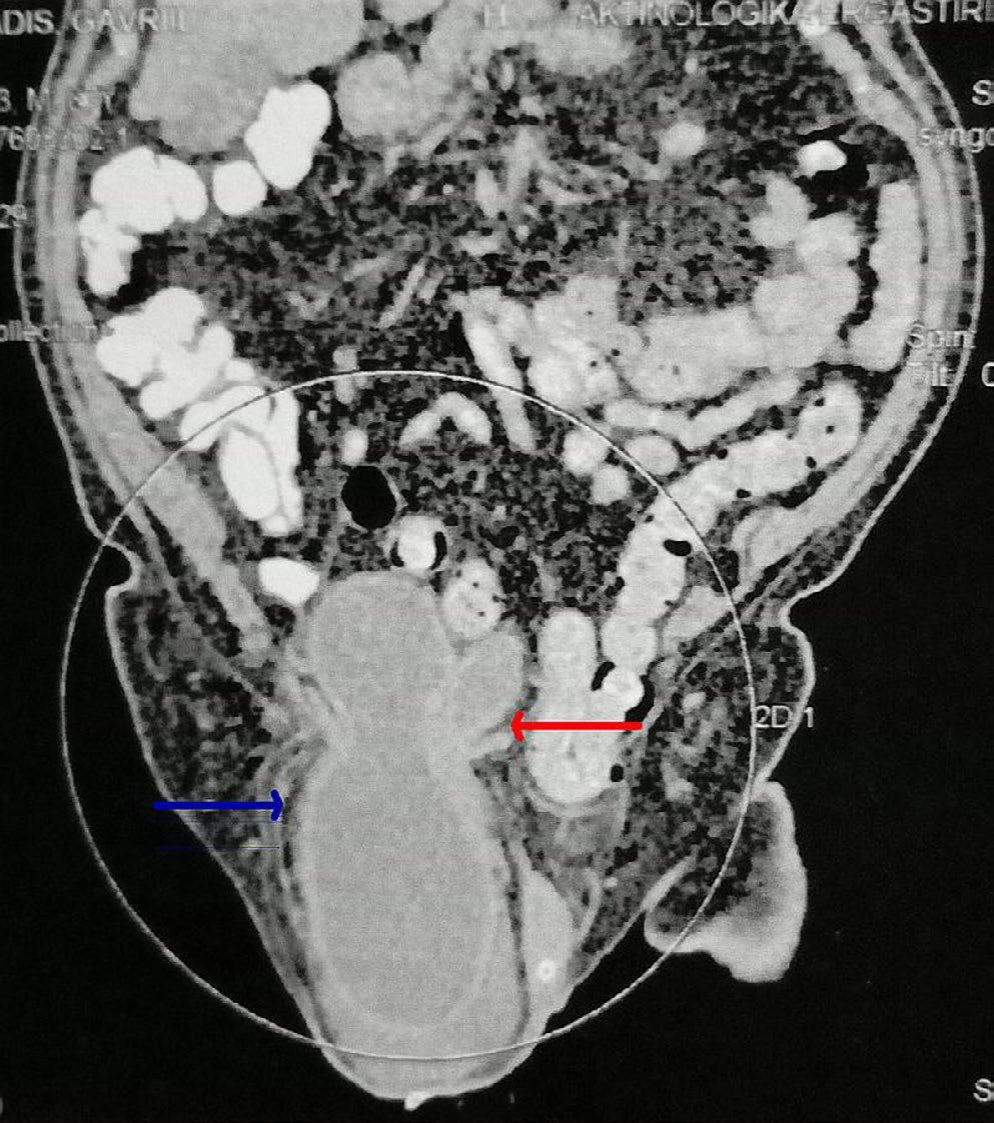
CT showed the bladder apex, body, and fundus (blue arrow) along with the trigone (red arrow) protruding into the hernia sac without herniation of bowel loops

## 1 QUIZ QUESTION: WHICH WAS THE CAUSE OF OBSTRUCTIVE UROPATHY?

Bladder apex, body, and fundus (blue arrow) along with the trigone (red arrow) protruded into the hernia sac without herniation of bowel loops, as shown in Figure 1. The bladder hernia entrained the trigone beyond the hernia neck into the inguinal canal leading to ureterovesical junction compression. The patient submitted to open mesh‐plug hernioplasty with immediate significant improvement in obstructive uropathy. Cystoscopy also performed which revealed no other cause of ureteral obstruction.

Inguinal bladder hernia is rare with an incidence of 1%–3% of all inguinal hernias. Pathophysiology involves urinary bladder outlet obstruction leading to chronic distention and atony along with abdominal wall weakness.^1^ Standard treatment is open reduction of the herniated urinary bladder followed by herniorrhaphy; bladder resection is rarely indicated in cases of bladder wall necrosis, tight hernia neck preventing reduction and presence of malignancy.^2^

## CONFLICT OF INTEREST

None declared.

## AUTHOR CONTRIBUTIONS

All authors equally accessed the data and contributed to the preparation of the manuscript. BKA and HA were equally responsible for making and performing treatment decisions. HA reviewed the manuscript for critical intellectual content and had the final approval.

## STATEMENT OF HUMAN AND ANIMAL RIGHTS

The present article does not contain any studies with human or animal subjects performed by any of the authors.

## INFORMED CONSENT

Informed consent was obtained from the patient.

## Data Availability

Data sharing is not applicable to this article as no datasets were generated or analyzed during the current study.
